# Coupled plasma filtration adsorption for the treatment of sepsis or septic shock: a systematic review and meta-analysis

**DOI:** 10.1186/s12879-022-07689-5

**Published:** 2022-08-29

**Authors:** Yuting Li, Hongxiang Li, Jianxing Guo, Youquan Wang, Dong Zhang

**Affiliations:** Department of Intensive Care Unit, The First Hospital of Jilin University, Changchun, 130021 Jilin China

**Keywords:** Coupled plasma filtration adsorption, Sepsis, Septic shock, Mortality, Meta-analysis

## Abstract

**Background:**

The effect of coupled plasma filtration adsorption (CPFA) for the treatment of sepsis or septic shock is controversial. A systematic review and meta-analysis was performed to evaluate the impact of CPFA on all-cause mortality in patients with sepsis or septic shock.

**Methods:**

We searched the PubMed, Cochrane, and Embase databases for randomized controlled trials (RCTs) and cohort studies from inception to the 1st of May 2022. We included studies involving patients (˃ 14 years) with sepsis or septic shock. All authors reported our primary outcome of all-cause mortality (hospital mortality, 28-day mortality or 30-day mortality). Results were expressed as odds ratio (OR) with accompanying 95% confidence interval (CI).

**Results:**

Six studies including 537 patients were included. The primary outcome of this meta-analysis showed that the all-cause mortality was about 54.2% (119/243 in the CPFA group and 172/294 in the control group). There was no statistically significant difference in the all-cause mortality between two groups (odds ratio [OR] = 0.75; 95% CI 0.53 to 1.06; P = 0.11; Chi^2^ = 14.04; I^2^ = 64%).

**Conclusions:**

The treatment of CPFA failed to decrease all-cause mortality of sepsis or septic shock patients. Further large-scale randomized controlled trials (RCTs) evaluating the ability of this therapy to improve clinical outcomes are still required to confirm these results.

**Supplementary Information:**

The online version contains supplementary material available at 10.1186/s12879-022-07689-5.

## Key messages

- The treatment of CPFA failed to decrease all-cause mortality of sepsis or septic shock patients.
- Potential drawbacks of this technique are the unexpected elimination of antibiotics, worsened procoagulant state and oxidative stress, expensive cost.
- Further rigorous investigation defining both the efficacy and safety of this otherwise promising hemopurification method on sepsis or septic shock is necessary.

**Supplementary Information:**

The online version contains supplementary material available at 10.1186/s12879-022-07689-5.

## Background

Sepsis is still a leading cause of mortality in intensive care unit (ICU) patients, mortality of sepsis and septic shock remains incredibly high, ranging between 20 and 40%, depending on the severity of illness [1, 2]. The pathophysiology of sepsis and septic shock is only partly understood, circulating pro-inflammatory and anti-inflammatory mediators appear to participate in the complex cascade of events, which leads to cell and organ dysfunction and, in many cases, death [3, 4]. A systemic inflammatory response with massive cytokine and inflammatory mediator release and the activation of coagulation and complement systems can be induced by the endotoxin of Gram-negative bacteria, which is one of the key triggers of sepsis.

Sepsis or septic shock mainly involves immune cell dysfunction and mediator dysregulation in response to an infection [5]. Terms such as “cell hyporesponsiveness” or “immunoparalysis” have been used to illustrate the inability of cells to respond to lipopolysaccharide (LPS) stimuli ex vivo due to overproduction of anti-inflammatory cytokines [6–9]. Evidence has been accumulated that severe bacterial infections and septic shock are associated with increased levels of plasma cytokines such as tumor necrosis factor-α (TNF-α) and interleukins (IL)-1 [10]. These inflammatory mediators are important for the antimicrobial response to local body. However, excessive release of the body and overproduction lead to the diffuse tissue injury and multiple organ dysfunction syndrome (MODS) [11]. Therefore, extracorporeal blood purification therapies have been proposed for patients with sepsis in order to improve outcomes since these therapies can alter the host inflammatory response by non-selective removal of inflammatory mediators or bacterial products or both [12].

Theoretically, extracorporeal therapies can be used to remove septic mediators from the bloodstream of critically ill patients [13], coupled plasma filtration adsorption (CPFA) is one such technology. CPFA is an extracorporeal blood purification treatment, which combines a first stage of plasma separation and adsorption of cytokines, inflammatory mediators and/or toxins, followed by a second stage of haemofiltration for volume control and removal of small water-soluble mediators [14]. CPFA was originally developed as a treatment for sepsis in the mid-1990s to address the need to remove cytokines and inflammatory mediators that are not easily or effectively removed by conventional extracorporeal methods (plasma exchange, haemodiafiltration, haemodialysis) [10].

Several studies have observed an improvement in haemodynamic parameters with CPFA in septic shock patients [15, 16]. However, the effect on mortality is still in controversy. Therefore, we conducted a meta-analysis which extracted results from published randomized controlled trials (RCTs) and cohort studies to evaluate the impact of CPFA on mortality in patients with sepsis or septic shock.

## Methods

This systematic review and meta-analysis is reported according to the updated Preferred Reporting Items for Systematic Reviews and Meta-Analyses (PRISMA) guidelines [17]. Ethical approval was not necessary for this study because it was a review of the published literature.

### Search strategy

**Fig. 1:**
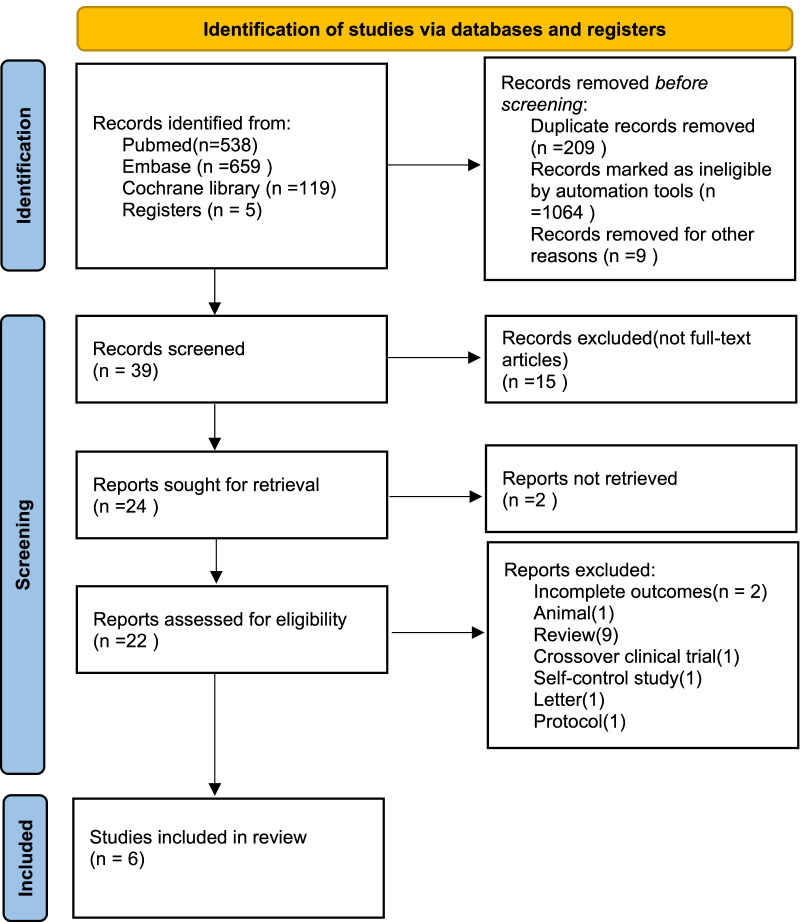
Flow chart of literature selection

We searched the PubMed, Embase databases and Cochrane Library for studies from inception to the 1st of May 2022 using the following search terms: coupled plasma filtration adsorption, coupled plasma filtration and adsorption, coupled plasma filtration with adsorption, CPFA, plasma adsorption, blood purification, hemoadsorption, sepsis, septic shock. The search was slightly adjusted according to the requirements of the different databases. The authors’ personal files and reference lists of relevant review articles were also reviewed. The search strategy for each database is showed in Additional file 1. The flow chart of the search strategies is summarized in Fig. 1.

### Types of outcome measures

The primary outcome was all-cause mortality, all-cause mortality included hospital mortality, 28-day mortality and 30-day mortality. Weighted means were calculated based on the number of patients in each study.

### Study selection

The inclusion criteria were as follows: (1) RCTs as well as prospective and retrospective cohort studies; (2) patients (˃ 14 years) with a diagnosis of sepsis or septic shock; (3) all authors reported our primary outcome of all-cause mortality; (4) clearly comparing CPFA group versus control group with clinically relevant outcomes. We excluded studies without clear comparisons of the outcomes. In addition, we excluded review articles and studies about pediatric or animal.

### Quality assessment

**Fig. 2:**
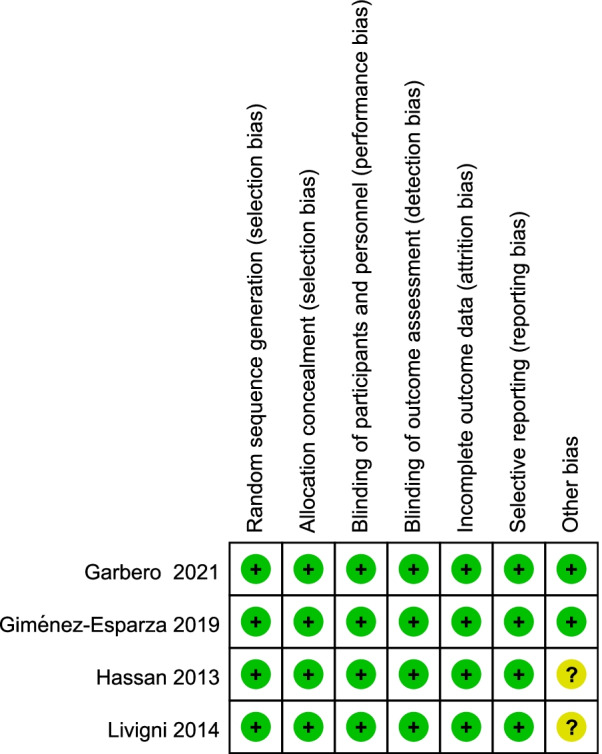
Risk of bias summary

**Fig. 3:**
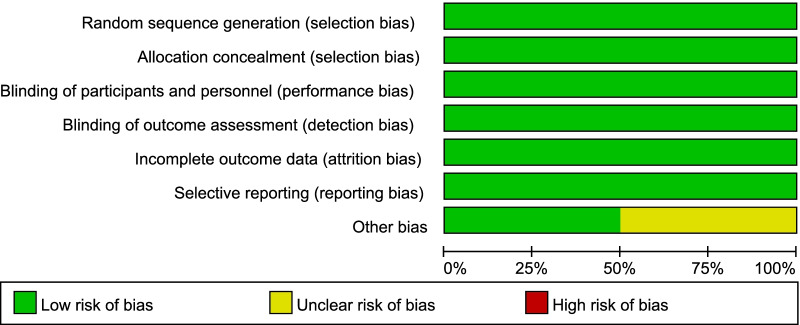
Risk of bias graph

Two reviewers (Yuting Li and Hongxiang Li) independently performed quality assessment. The quality of studies was assessed using the Cochrane Collaboration’s tool for RCTs [18], and the Newcastle–Ottawa Scale (NOS) was used for cohort studies [19]. The specific elements to minimize bias of RCTs were: (1) randomization sequence (selection bias), (2) allocation concealment (selection bias), (3) blinding of study personnel and participants (performance bias), (4) blinding of outcome assessors (performance bias), (5) complete reporting of data without arbitrarily excluded patients and with low to minimal loss to follow-up (attrition bias), (6) selective reporting bias, and (7) other sources of bias. Satisfactory performance, unclear performance, and unsatisfactory performance of each domain from the tool is denoted by green, yellow, and red color respectively. The risk of bias summary for included RCTs is presented in Fig. 2, the risk of bias graph for included RCTs is presented in Fig. 3.

NOS allocates a maximum of 9 points according to the quality of the selection, comparability, and outcomes of the cohort study populations. Study quality was defined as poor (0–3), fair (4–6) or good (7–9). The quality of the included cohort studies is presented in Table 1.

**Table 1:** Quality of the included cohort studies (The Newcastle–Ottawa Scale)

Study	Selection				Comparability	Outcome			Total score
	Representativeness of the exposed cohort	Selection of the non exposed cohort	Ascertainment of exposure	Demonstration that outcome of interest was not present at start of study	Comparability of cohorts on the basis of the design or analysis	Assessment of outcome	Was follow-up long enough for outcomes to occur	Adequacy of follow up of cohorts	
Yaroustovsky [23]	☆	☆	☆	☆	☆☆	☆	☆	☆	9
Mariano [25]	☆	☆	☆	☆	☆☆	☆	☆	☆	9

Note: a star represents 1 point, with a full score of 9 points

### Statistical analysis

Statistical analyses were performed using Review Manager Version 5.3 (RevMan, The Cochrane Collaboration, Oxford, United Kingdom). Odds ratio (OR) with 95% confidence intervals (CI) was calculated for dichotomous variables. A random-effects model was used to pool studies with significant heterogeneity, as determined by the Chi-squared test (P < 0.10) and inconsistency index (I^2^ ≥ 50%) [20]. A P-value < 0.05 was set as the threshold of statistical significance. To reduce bias, we performed a subgroup analysis of RCTs and cohort studies.

## Result

### Study characteristics

The search strategy identified 1316 studies, and the data were from four RCTs and two cohort studies comprising 537 patients (Table 2) [21–26]. The characteristics of the included studies are shown in Table 2. A total of six eligible studies were published between 2013 and 2021. Among these studies, one study was conducted in Malaysia, one study was conducted in Egypt, one study was conducted in Spain and three studies were conducted in Italy. Three of these studies were single-center studies and others were multicenter studies.

**Table 2:** The basic characteristics of studies included in meta-analysis

Author	Year	Country	Study period	Study design	No. of patients		
					Total	CPFA	Control
Hassan [21]	2013	Malaysia	Aug. 2011–Jan. 2012	Single center, RCT	23	11	12
Livigni [22]	2014	Italy	Jan. 2007–Nov. 2010	Multicenter, RCT	184	91	93
Yaroustovsky [23]	2015	Egypt	Jan. 2010–Jun. 2014	Single center, prospective cohort study	40	20	20
Giménez-Esparza [24]	2019	Spain	–	Multicenter, RCT	49	19	30
Mariano [25]	2020	Italy	Jan. 2001–Dec. 2007	Single center, retrospective cohort study	126	39	87
Garbero [26]	2021	Italy	May. 2015–Oct. 2017	Multicenter, RCT	115	63	52

### Primary outcome

**Fig. 4:**
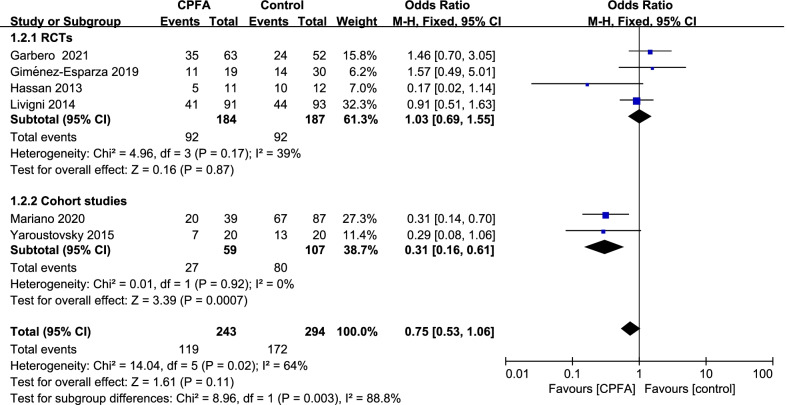
Forest plot for all-cause mortality

**Fig. 5:**
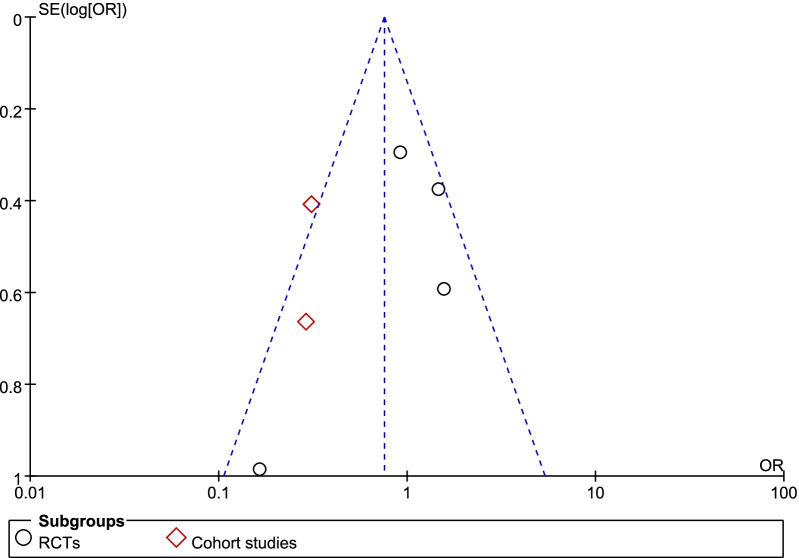
Funnel plot for all-cause mortality

A total of five studies including 537 patients were included, and the all-cause mortality was about 54.2% (119/243 in the CPFA group and 172/294 in the control group). There was no statistically significant difference in the all-cause mortality between two groups (odds ratio [OR] = 0.75;95% CI 0.53 to 1.06; P = 0.11; Chi^2^ = 14.04; I^2^ = 64%) (Fig. 4). A funnel plot was used to assess the publication bias (Fig. 5).

## Discussion

Sepsis is one of the main causes of death in critically ill patients worldwide, and in many cases it is associated with renal and/or other organ failure. However, we do not have a unique efficient therapy to reduce this extremely high mortality rate. Both pro-inflammatory and anti-inflammatory mediators participate in the pathogenesis of sepsis and explain the failure of specific therapies to improve survival. Continuous extracorporeal therapies have been proposed as a therapeutic option in sepsis [27]. One of the emerging treatments in patients with sepsis and septic shock is CPFA.CPFA is a technique that separates plasma from the blood using a plasma filter. The plasma is then passed through a synthetic resin cartridge and returned to the blood. A second blood filter is used to remove excess fluid and small molecular weight toxins [28]. The nonselective removal of inflammatory mediators is achieved by hydrophobic styrene resin, which has high affinity and capacity for many cytokines and mediators [29]. In vitro studies have demonstrated the efficacy of CPFA in adsorbing inflammatory mediators like IL-1β, IL-6, IL-8, IL-10, and TNF-α amongst others [27]. CPFA has also been shown to enhance early hemodynamic stability, reduce inotropic support requirement, and improve the immune response in septic patients [30]. However, these trials have so far failed to demonstrate any improvement in hard clinical outcomes.

Our systematic review and meta-analysis of six studies including 537 patients compared CPFA and control group in patients with sepsis or septic shock. We found that the overall all-cause mortality was about 54.2% and there was no statistically significant difference in the all-cause mortality between two groups. Guidelines, for example, state that ‘hemofiltration should not be used in patients with sepsis without renal indications unless ongoing studies provide positive results’ [31]. The role of plasma exchange remains equally controversial [32, 33]. The extracorporeal removal of septic mediators is not recommended in the 2016 edition of the Surviving Sepsis Campaign (SSC) due to the absence of large, randomized controlled trials demonstrating its efficacy [34]. Experimental study even showed that treatment with CPFA did not protect from progression of septic hypotension; failed to counteract the progressive alterations in microcirculatory perfusion, energy metabolism, and organ function; and even aggravated the sepsis-induced disturbances in coagulation and oxidative/nitrosative stress [29].

What are the implications of our meta-analysis’s results? Firstly, CPFA is a blood purification therapy aimed at modulating the host inflammatory response involved in sepsis pathogenesis. CPFA not only removes substances harmful to the body, but also removes beneficial substances. Piperacillin, tazobactam, and vancomycin, administered during CPFA, using the appropriate dosing regimens, achieved acceptable serum concentrations, despite adsorption on the resin cartridge [35]. However, a potential disadvantage of this technique is that it may accidentally eliminate other kinds of antibiotics. Any delay in receiving appropriate antibiotic therapy in severe sepsis or septic shock patients is associated with excess mortality [36–38]. Moreover, according to calculation, CPFA may removes 50% more antibiotics than does standard continuous renal replacement therapy, increasing the possibility of undertreatment. Increasing antibiotic clearance by adding the effect of at least 10 h’ renal replacement therapy to a well-functioning kidney could have caused treatment underdosing [26]. A significant dose–response effect of treated plasma on mortality was demonstrated in patients without severe renal failure. As a result, monitoring of antibiotics serum concentrations remains essential to avoid antibiotics underdosing. Secondly, even though previous studies have been promising, numerous questions, including the timing, duration, and frequency of these therapies in the clinical setting, remain unanswered. We hypothesize a connection to hemodynamic instability consequent on renal replacement therapy [39] that has been shown to increase mortality [40]. This instability may complicate the said therapy, especially when patients have not been fully stabilized, and may be related to early commencement of treatment(no more than 12 h from diagnosis) [26]. Thirdly, early treatment with CPFA failed to afford any protection against sepsis-mediated hemodynamic and physiological disturbances and tended to worsen procoagulant state and oxidative stress [29]. Fourthly, they did not take cost into account for each treatment. The cost of new sorbents may be one the main drawbacks in CPFA.

There are several limitations in our meta-analysis. First, the number of included studies is small. Further randomized clinical studies should be conducted in order to confirm the results. Second, many of the clinical outcomes such as ICU length of stay, hospital length of stay, hemodynamic parameters were not included in most of the studies examined in this meta-analysis. Therefore, we were unable to conduct a meta-analysis on secondary outcomes. Third, Organ dysfunction is also a very important clinical outcome. However, few included studies had showed this data. Fourth, although we had performed a subgroup analysis of RCTs and cohort studies, there was still substantial heterogeneity among the included studies. Very heterogeneous populations were included in both observational and randomized studies. In addition, inclusion/exclusion criteria and comorbidities were widely different among included studies which supposed a limitation to interpret results. Therefore, our findings should be interpreted with caution.

## Conclusion

In our systematic review and meta-analysis, the treatment of CPFA failed to decrease all-cause mortality of sepsis or septic shock patients. This result indicates that further rigorous investigation defining both the efficacy and safety of this otherwise promising hemopurification method on sepsis or septic shock is necessary. Further large-scale RCTs evaluating the ability of this therapy to improve clinical outcomes are still required to confirm these results.

## Supplementary Information

**Additional file 1.** Search strategy for each database.

## Data Availability

All data generated or analyzed during this study are included in this published article.

## Abbreviations

CPFA: Coupled plasma filtration adsorption
ICU: Intensive care unit
LPS: Lipopolysaccharide
TNF-α: Tumor necrosis factor-α
IL: Interleukins
MODS: Multiple organ dysfunction syndrome
RCTs: Randomized controlled trials
PRISMA: Preferred Reporting Items for Systematic Reviews and Meta-Analyses
NOS: Newcastle–Ottawa Scale
OR: Odds ratio
CI: Confidence interval
SSC: Surviving Sepsis Campaign
