# Supplementary material for: Coupled plasma filtration adsorption for the treatment of sepsis or septic shock: a systematic review and meta-analysis
Source: BMC Infect Dis. 2022 Aug 29;22:714. doi: 10.1186/s12879-022-07689-5 (PMC9422100; doi:10.1186/s12879-022-07689-5)
Supplement: Supplementary file 1 — Additional file 1. Search strategy for each database. [file 12879_2022_7689_MOESM1_ESM.docx]

**Additional table. Search strategy for each database**

| **Database** | **Search strategy** |
| --- | --- |
| Pubmed | #1 ((((((coupled plasma filtration adsorption[Title/Abstract]) OR (coupled plasma filtration and adsorption[Title/Abstract])) OR (coupled plasma filtration with adsorption[Title/Abstract])) OR (CPFA[Title/Abstract])) OR (plasma adsorption[Title/Abstract])) OR (blood purification[Title/Abstract])) OR (hemoadsorption[Title/Abstract]) |
|  | #2 ((((((((((((((((((((((((((((("Sepsis"[Mesh]) OR "Shock, Septic"[Mesh]) OR (Bloodstream Infection[Title/Abstract])) OR (Bloodstream Infections[Title/Abstract])) OR (Infection, Bloodstream[Title/Abstract])) OR (Pyemia[Title/Abstract])) OR (Pyemias[Title/Abstract])) OR (Pyohemia[Title/Abstract])) OR (Pyohemias[Title/Abstract])) OR (Pyaemia[Title/Abstract])) OR (Pyaemias[Title/Abstract])) OR (Septicemia[Title/Abstract])) OR (Septicemias[Title/Abstract])) OR (Poisoning, Blood[Title/Abstract])) OR (Blood Poisoning[Title/Abstract])) OR (Blood Poisonings[Title/Abstract])) OR (Poisonings, Blood[Title/Abstract])) OR (Severe Sepsis[Title/Abstract])) OR (Sepsis, Severe[Title/Abstract])) OR (Septic Shock[Title/Abstract])) OR (Shock, Toxic[Title/Abstract])) OR (Toxic Shock Syndrome[Title/Abstract])) OR (Shock Syndrome, Toxic[Title/Abstract])) OR (Toxic Shock Syndromes[Title/Abstract])) OR (Toxic Shock[Title/Abstract])) OR (Shock, Endotoxic[Title/Abstract])) OR (Endotoxin Shock[Title/Abstract])) OR (Endotoxin Shocks[Title/Abstract])) OR (Shock, Endotoxin[Title/Abstract])) OR (Shocks, Endotoxin[Title/Abstract]) |
|  | #3 #1 AND #2 |
| Embase | #1 'sepsis'/exp  #2 'septic shock'/exp  #3 'abdominal sepsis'/exp OR 'abdominal sepsis' OR (abdominal AND ('sepsis'/exp OR sepsis)) OR 'focal sepsis':ab,ti OR 'intraabdominal sepsis':ab,ti OR 'sepsis syndrome':ab,ti OR 'septic disease':ab,ti OR 'sepsis-associated hypotension':ab,ti OR 'septicaemic shock':ab,ti OR 'septicemic shock':ab,ti OR 'shock, septic':ab,ti  #4 'coupled plasma filtration adsorption'/exp OR 'coupled plasma filtration adsorption' OR (coupled AND ('plasma'/exp OR plasma) AND ('filtration'/exp OR filtration) AND ('adsorption'/exp OR adsorption)) OR ('coupled plasma filtration':ab,ti AND adsorption:ab,ti) OR 'coupled plasma filtration with adsorption':ab,ti OR cpfa:ab,ti OR 'plasma adsorption':ab,ti OR 'blood purification':ab,ti OR hemoadsorption:ab,ti  #5 #1 OR #2 OR #3  #6 #4 AND #5 |
| Cochrane  library | #1 MeSH descriptor: [Sepsis] explode all trees  #2 (“Pyemias” OR “Pyaemia” OR “Pyemia” OR “Pyohemias” OR “Pyohemia” OR “Pyaemias” OR “Blood Poisonings” OR “Blood Poisoning” OR “Poisoning, Blood” OR “Poisonings, Blood” OR “Septicemia” OR “Septicemias” OR “Bloodstream Infection” OR “Infection” OR “Bloodstream” OR “Bloodstream Infections” OR “Severe Sepsis” OR “Sepsis, Severe”):ti,ab,kw  #3 MeSH descriptor: [Shock, Septic] explode all trees  #4 (“Shock, Endotoxic” OR “Shocks, Endotoxin” OR “Endotoxin Shocks” OR “Shock, Endotoxin” OR “Endotoxin Shock” OR “Septic Shock” OR “Toxic Shock” OR “Toxic Shock Syndromes” OR “Shock, Toxic” OR “Toxic Shock Syndrome” OR “Shock Syndrome, Toxic”):ti,ab,kw  #5 (“coupled plasma filtration adsorption” OR “coupled plasma filtration and adsorption” OR “coupled plasma filtration with adsorption” OR “CPFA” OR “plasma adsorption” OR “blood purification” OR “hemoadsorption”):ti,ab,kw  #6 #1 or #2 or #3 or #4  #7 #5 and #6 |
